# Translational Implications of Dysregulated Pathways and microRNA Regulation in Quadruple-Negative Breast Cancer

**DOI:** 10.3390/biomedicines10020366

**Published:** 2022-02-02

**Authors:** Amal Qattan, Taher Al-Tweigeri, Kausar Suleman

**Affiliations:** 1 Translational Cancer Research Section, Department of Molecular Oncology, King Faisal Specialist Hospital and Research Centre, Riyadh 11211, Saudi Arabia; 2 Department of Medical Oncology, Oncology Centre, King Faisal Specialist Hospital and Research Centre, Riyadh 11211, Saudi Arabia; ttwegieri@kfshrc.edu.sa (T.A.-T.); ksuleman@kfshrc.edu.sa (K.S.)

**Keywords:** quadruple-negative breast cancer (QNBC), TNBC, androgen receptor, microRNA

## Abstract

Triple-negative breast cancers (HER2−, ER−, PR−) continue to present a unique treatment challenge and carry unfavorable prognoses. The elucidation of novel therapeutic targets has necessitated the re-evaluation of stratification approaches to best predict prognosis, treatment response and theranostic and prognostic markers. Androgen receptor expression and function have important implications on proliferation, tumor progression, immunity and molecular signaling in breast cancer. Accordingly, there has been increasing support for classification of androgen receptor-negative triple-negative breast cancer or quadruple-negative breast cancer (QNBC). QNBC has unique molecular, signaling and expression regulation profiles, particularly those affected by microRNA regulatory networks. microRNAs are now known to regulate AR-related targets and pathways that are dysregulated in QNBC, including immune checkpoint inhibitors (ICIs), SKP2, EN1, ACSL4 and EGFR. In this review, we explore and define the QNBC tumor subtype, its molecular and clinical distinctions from other subtypes, miRNA dysregulation and function in QNBC, and knowledge gaps in the field. Potential insights into clinical and translational implications of these dysregulated networks in QNBC are discussed.

## 1. Introduction

Breast cancers lacking epidermal growth factor receptor 2 (HER2), estrogen receptor and progesterone receptor, are termed triple-negative breast cancers (TNBC) and are distinguished because of a lack of response to therapies targeting these receptors. TNBC is heterogeneous at genomic, transcriptomic levels, showing varying clinical and pathological presentation genetic susceptibility factors and sensitivities for chemotherapeutics [1]. As such, the use of biomarkers and subtype classification of TNBC lacks standardization. Partly because of this heterogeneity and lack of focus on targetable molecules or pathways, there is a lack of effective targeted approaches to this disease [2]. However, in recent years, androgen receptor (AR) has emerged as a target in TNBC [3,4,5]. While AR is expressed in the majority of breast cancers (70–90%), expression is less prevalent in TNBC (10–50%) [5]. AR regulates multiple molecular pathways that are related to tumorigenesis and tumor progression by both transcriptional and non-transcriptional mechanisms [5]. Receptor hormone complexes enter the nucleus, enter a complex with co-activators and activate target genes. Activated AR binding to estrogen responsive elements may competitively inhibit estrogen-responsive gene expression. Alternatively, AR can interact with cytoplasmic phosphoinositide 3-kinase (PI3K), Ras or Src proteins to promote proliferative and survival signaling. These activities result in distinct molecular and phenotypic profiles in AR-positive TNBC (AR+TNBC) compared with AR-negative TNBC or quadruple-negative breast cancer (QNBC). Examples include increased PI3K signaling and mutation, epithelial to mesenchymal transition (EMT), cell-cycle deregulation, cytotoxic T-cell recruitment and aberrant DNA repair [5]. These distinct profiles provide unique opportunities for targeted therapeutic use and development for QNBC. In addition, AR itself may be targeted therapeutically. Possibly owing to the estrogen response antagonism mentioned above, there is in vitro and preclinical evidence for both agonism and antagonism of AR having therapeutic effects in breast cancer [4].

Differential expression profiles and pathway regulation in breast cancer and TNBC in particular are integrated with the expression and regulation of transcriptional profiles by microRNAs (miRNA) [6]. Importantly in the context of AR-positive breast cancers and QNBC, recent efforts have identified androgen-responsive miRNAs, the expression of specific miRNAs and miRNA expression profiles that are associated with AR expression in breast cancer [7,8,9]. Further, evidence suggests that androgen regulation of miRNAs may be required for proliferative signaling mediated by androgens in breast cancer [8]. While these discoveries have important implications on the targeting of signaling pathways, molecular targets and miRNAs themselves in breast cancer and QNBC, evidence for strong associations of specific miRNAs and downstream pathways are newly emergent and require focused investigation to delineate new targets and treatment options for AR-positive versus AR-negative disease. Herein, we review the QNBC tumor subtype, molecular and clinical distinction from other subtypes, miRNA dysregulation and function in QNBC and knowledge gaps in the field.

## 2. Definition and Clinical Distinctions of QNBC Compared with TNBC and Other Subtypes of Breast Cancer

TNBC has classically been divided into basal-like subtypes (BL1 and BL2), mesenchymal (M) and luminal androgen receptor (LAR) subtypes, and in some systems, according to immunosuppressive versus immune active phenotypes [5,10,11,12,13]. TNBC comprises 15–20% of breast cancers and carries the lowest survival rate of all breast cancer subtypes and an increased risk of distant recurrence [3,12]. TNBC carries increased metastasis, an increased relapse rate and a younger age of onset [12]. TNBC is difficult to treat because of poor differentiation, molecular heterogeneity and metastasis, and receptor negativity eliminates response to hormone or anti-Her2 therapies [12,14]. Studies have reported decreased AR expression in TNBC compared with other types, with between 10 and 50% of TNBC tumors lacking AR expression **[5]**. There has been increased attention given to the utility of reclassifying TNBC tumors lacking AR expression as QNBC [3,5,15,16]. AR positivity in TNBC has been found to be positively associated with both overall and disease-free survival (OS and DFS, respectively) [3,17,18,19,20]. Meta-analysis showed that AR-positive TNBC patients had more metastasis to lymph nodes and lower tumor grade [21]. This and other meta-studies showed significantly better DFS with AR expression, some also demonstrating significantly increased OS [9,20] and others being non-significant for this outcome in TNBC [21]. In a study of the clinicopathologic features of TNBC related to AR and EGFR positivity by Astvatsaturyan et al., AR+, EGFR− disease, representing the LAR subtype, carried the best prognosis, and AR−, EGFR+ disease, representing the basal subtype, carried the worst prognosis with regard to DFS [22]. EGFR expression (≥15%) in this study been found to be present in 30% of TNBC overall, and in 35% of AR- TNBC. This is in agreement with another study showing EGFR overexpressed in 30% of TNBC tumors [23]. Potentially related to disease outcomes, AR expression in TNBC was found to be inversely correlated with proliferative marker Ki-67 expression and lympho-vascular invasion [18]. When included as a classification, QNBC has been described as having the worst prognosis among breast cancer subtypes [16]. These findings suggest that QNBC is a more formidable clinical challenge than TNBC with poor prognosis and increased potential for an invasive phenotype.

Regardless of some controversy regarding the prognostic significance of AR expression in TNBC [15], the primary benefit of classifying QNBC separately from TNBC is that TNBC with AR expression may be effectively treated using AR-targeted therapies [3,15]. Aside from AR-targeted therapies, there have been no effective targeted therapies for TNBC. Anti-androgen therapy has been found to be effective in inhibiting proliferation in TNBC cells and to benefit patients with LAR-subtype TNBC [24,25,26]. To this point, Gucalp et al. demonstrated a 19% response rate to the AR inhibitor bicalutamide in AR-positive hormone receptor-negative breast cancer patients [26]. QNBC, however, lacks all known receptor targets that have been exploited for cancer therapy. Regarding non-targeted therapy, AR expression has also been found to be positively associated with and predictive of response to neoadjuvant chemotherapy in breast cancer [27,28]. Therefore, in addition to carrying a relatively less favorable prognosis than AR-positive TNBC, QNBC presents an outstanding challenge in finding and implementing targeted therapies.

## 3. Significance of Androgen Receptor Expression and Function in Breast Cancer

AR is a 110 kDa protein residing in the cytoplasm with zinc finger DNA-binding, transcriptional regulation and ligand-binding capabilities [29]. Within the cytoplasm, AR is chaperoned by heat shock proteins HSP10 and HSP90. Androgen steroids serve as ligands for AR, altering the conformation of AR and releasing chaperone proteins upon binding. This conformational change allows entry into the nucleus, dimerization and binding to androgen-response elements within genomic DNA. Binding of AR complexed with co-activator proteins to these elements drives transcription of target genes. Through this classical pathway of AR action and other mechanisms discussed in Section 4, AR in TNBC functions to alter proliferation, cell-cycle, epithelial-to-mesenchymal transition, angiogenesis and immunity [5].

Proliferation of tumor cells is generally thought to be increased by AR with evidence of cell-cycle regulation downstream of various androgen-responsive transcriptional activities [30]. AR-ligand dihydrotestosterone (DHT) increased proliferation and decreased apoptosis in mesenchymal TNBC, which was reversed by anti-androgen receptor treatment with bicalutamide [31].

AR expression in TNBC may also affect epithelial-to-mesenchymal transition (EMT) leading to altered invasion and metastasis. To this point, nuclear AR was found to be inversely associated with E-cadherin expression and positively associated with increased tumor grade, mesenchymal morphology, metastasis, recurrence and with poor DFS and OS in TNBC [32,33]. Liu et al. demonstrated an AR-binding site in the E-cadherin gene, the expression of which characterizes an epithelial phenotype, suggesting a direct regulation of EMT through E-cadherin repression [33].

While evidence for angiogenic activity of AR in breast cancer is sparse, regulation of angiogenesis by AR in prostate cancer is implicated through interactions with epigenetic and transcriptional co-activation factors to regulate VEGF, and synergy between anti-androgenic and anti-VEGF therapies [34].

Immune killing of TNBC can also be increased through androgen deprivation therapy using enzalutamide or abiraterone, although this effect was shown to be partially independent of detection of AR expression [35].

Androgen receptor signaling has the potential to regulate DNA repair in QNBC as anti-androgen therapy has been shown to downregulate DNA repair genes that are direct transcriptional targets of AR [36]. In addition, androgen enhanced DNA repair following ionizing radiation has been shown. This enhancement was ablated by anti-androgen treatment, which also resulted in decreased non-homologous end-joining.

Given that many of the phenotypic effects of AR are pro-tumorigenic and pro-tumor progression, much remains to be discovered regarding mechanisms by which AR negative TNBCs or QNBCs generally have poorer prognoses than AR-positive TNBCs.

## 4. Molecular Pathways Dysregulated Specifically in QNBC

Subtypes of TNBC, including basal-like, mesenchymal, LAR, and immunomodulatory, are characterized by distinct molecular pathways and profiles [37] as outlined in Table 1. Androgen receptor activity is known to antagonize estrogen receptor activity with a high AR:ER ratio being directly correlated with poorer prognosis in ER-positive breast cancer [38] and increased expression of proliferative markers [39]. However, this ratio is not relevant to TNBC, which lacks ER expression. The distinction between TNBC and QNBC is weighed more heavily on other molecular mechanisms of AR action, including those affecting cell-cycle regulation, apoptosis and proliferative signaling. Activated AR in complex with FOXA1 can drive transcription of Myc, Wnt7B and Her3, representing pathways directing tumor survival, stemness and proliferation [38]. Multiple molecular mechanisms of AR activity on proliferation and cell-cycle regulation have been elucidated. Cell cycle regulators Cyclin D1 and p21 are direct targets of repression and activation by AR, respectively [38]. Balance between these two opposing AR targets may dictate promotion versus inhibition of proliferation by the presence of AR signaling. AR expression has been found to decrease proliferation of TNBC cells, decrease expression of cell-cycle regulator Cyclin D1 and increase that of p73 and p21, while AR-ligand DHT increased their expression [31]. AR-mediated downregulation of G-protein coupled estrogen receptor (GPCR) expression was also found to promote proliferation in TNBC cells [30]. Androgen receptor transcriptional activation of PTEN and KILLIN (KLLN), which feeds into p53 and p73 activity, represents apoptotic signaling pathways regulation that may contribute to tumor suppression [5,38]. Lack of AR regulation of these pathways in QNBC and the global regulatory networks surrounding those pathways may represent therapeutic targeting opportunities. 

**Figure 1:**
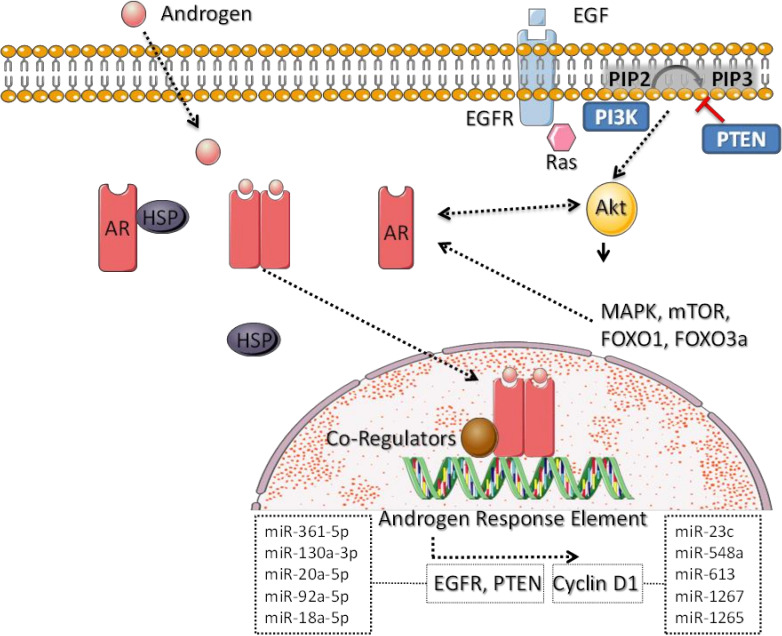
TNBC-relevant androgen receptor signaling and miRNA regulation. Cytoplasmic androgen receptors (AR), chaperoned by heat-shock proteins (HSPs), are activated to dimerize and cross the nuclear membrane upon engagement with androgen ligand. Thereafter, activated AR dimers complex with co-regulators to regulate androgen response elements within target genes, including EGFR and Cyclin D1. Growth factor signaling, particularly that involving the AKT signaling axis, promotes crosstalk and feedback with AR signaling as described in further detail in the text.

In addition to direct activity through androgen-response elements, non-DNA-binding mechanisms of AR function exist [40,41], including activation of second messengers, including Akt and the downstream MAPK pathway, mTOR and FOXO1, and transrepression of other transcriptional regulators, such as activator protein 1 (AP1) [40,41,42]. Luminal androgen receptor-expressing (LAR) TNBC patient-derived xenografts were found to be enriched for AKT1 and PIK3CA mutations and FGFR1 amplifications compared with other TNBC subtypes [43]. There is a direct interaction of activated AR with PI3K that promotes generation of PIP3 and activation of AKT, and AKT substrate FOXO3a in turn promotes expression of AR [44]. Meanwhile, PTEN, a negative regulator of PIP3 signaling, is transcriptionally activated by AR in breast cancer [45]. Such cross-talk between AR, growth factor receptors and the AKT signaling axis presents a potentially targetable signaling network. Figure 1 provides an illustration of AR signaling pathways related to TNBC and miRNAs regulating relevant pathways as identified in the literature and by network analysis, described in detail in Section 6 and Section 7.

In contrast to findings associated with increased EMT with AR expression in TNBC [32], expression levels of EMT-associated genes (SNAI2, TCF7L2, ACTA2, VIM, and CAV1) have been found to be decreased in LAR TNBC relative to other TNBCs using patient-derived xenografts [43]. Caveats to this finding include possible alterations in xenografted models and limitation to the LAR subtype, which is exclusive of mesenchymal types. The LAR subtype is characterized by both AR expression and activation of hormone signaling pathways, such as steroid synthesis, androgen and estrogen metabolism and porphyrin metabolism [24]. QNBC predominantly exhibits a basal-like expression profile [46,47].

## 5. Molecular Profiles Observed in QNBC

QNBC tumors display distinct molecular profiles including that of transcriptional expression as shown by a study on African American women showing enriched classical basal-like and immune subtype signatures in QNBC [48]. In this cohort, expression levels of E2F1, NFKBIL2, CCL2, TGFB3, CEBPB, PDK1, IL12RB2, IL2RA and SOS1 were associated with AR expression within the broader classification of TNBC. The magnitude of these differences was somewhat dependent on race for some of these genes. The differential molecular profile of QNBC presents some potential therapeutic targets, such as S-phase kinase-associated protein 2 (SKP2), EGFR, Engrailed 1 (EN1) and acyl-CoA synthase 4 (ACSL4) [16,46]. EGFR is a well-known targetable pro-tumorigenic growth factor that is commonly expressed in TNBC and especially QNBC [46,49]. SKP2 is an ubiquitin ligase component that contributes to the degradation of tumor suppressors p21, p27 and p57 and is expressed during S-phase to promote DNA replication. ACSL4 catalyzes long chain fatty acid activation for the support of metabolic processes, and its expression in breast cancer is inversely correlated with the expression of AR in addition to that of ER, PR and HER2 [46,50,51]. While normally expressed in neural tissues as a transcription factor, among breast cancers, EN1 is only overexpressed in basal-like tumors [16,52]. EN1 expression is associated with brain metastasis and low overall survival within TNBCs [16,53]. Targeted therapies, including nanoparticles with EN1-inhibiting peptides, have been developed and have shown significant TNBC growth inhibition in vitro and in vivo without toxicity [54].

Broad regulation of transcriptional, and subsequently, molecular pathways by miRNAs is known to be important to the understanding of breast cancer pathobiology and markers of the disease. As such, we continue with a detailed review of miRNA regulators of the above pathways, known miRNA dysregulation in QNBC, clinical and translational implications of miRNA regulation in QNBC and unknowns in the field.

## 6. Dysregulated miRNAs Related to QNBC and Their Molecular Functions

Since little is known regarding QNBC-specific miRNA regulatory networks, much of the data driving hypotheses for miRNA regulation affecting QNBC pathobiology are extrapolated from the study of TNBC, AR function in breast cancer, and from other cancers. Integration of this existing knowledge, as in this review, provides a jumping-off point for the study of miRNA dysregulation in QNBC. Among the potentially targetable molecular pathways that are known to be altered in QNBC as described above in Section 4, ACSL4 has been found to be regulated by dysregulated miRNAs in cancer. MiR-211-5p was found to act as a tumor suppressor in hepatocellular carcinoma through direct targeting of ACSL4 [55]. This miRNA was identified by screening using Gene Expression Omnibus datasets (GEO) and evaluated for prognostic association by Kaplan–Meier analysis (KM), revealing that decreased miR-211-5p was associated with poor overall survival (OS). Restoration of miR-211-5p expression in vitro suppressed migration, invasion and proliferation. Suppression of the malignant phenotype by miR-211-5p was found to be through inhibition of ACSL4 expression, and re-expression of ACSL4 restored a malignant phenotype. MiR-133a has been found to be down-regulated in receptor negative breast cancer cells and tissue and to directly target the 3’ UTR of EGFR [56]. Exogenous re-expression of miR-133a in these cells reduced EGFR expression, AKT phosphorylation and nuclear translocation of phosphorylated AKT. Therefore, miR-133a may act as a tumor suppressor through inhibition of the EGFR-AKT axis in breast cancers with EGFR expression, which is a hallmark of QNBC. Similarly, miR-361-5p was recently found to inhibit migration and invasion in TNBC through the EGFR-AKT pathway by direct targeting of Required for Cell Differentiation 1 (RQCD1), which facilitates AKT activation by EGFR [57]. RQCD1 is frequently upregulated and miR-361-5p frequently downregulated in breast cancer, which was confirmed in TNBC in this study, although this study stopped short of establishing a direct mechanistic link between RQCD1 regulation by miR-361-5p and EGFR-AKT axis regulation.

**Figure 2:**
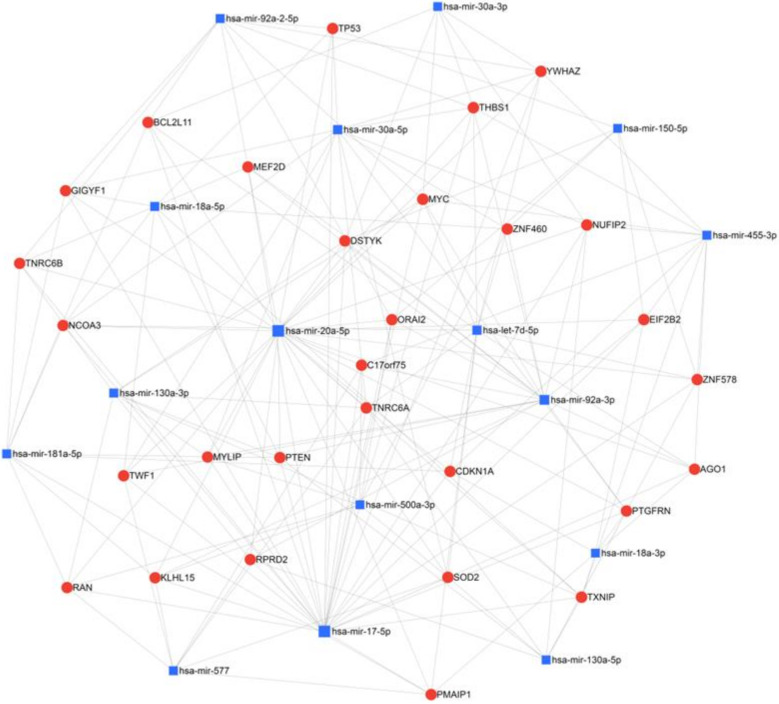
Network analysis of miRNAs found to be upregulated in QNBC in African American women by Angajala et al. using miRNet 2.0 with minimum network filtering. The miRNAs identified by this study and the genes they are known to regulate were analyzed for relatedness and common nodes, revealing prevalent regulation networks and regulated genes in this population.

Other miRNAs that are specifically dysregulated in QNBC have been recently discovered. QNBC was found to exhibit increased expression of miR-135b, which correlated with AR-negativity among TNBCs [58]. Differentially expressed miRNAs were screened by array analysis and confirmed by RT-qPCR in tumors of both basal-like and non-basal-like (QNBC) TNBC subtypes. MiR-135b was suggested to promote QNBC pathogenesis and to be associated with the targeting of TGF-β, WNT and ERBB signaling pathways. MiR-135b expression was strongly and positively correlated with a high proliferative index. A recent study of African American women with QNBC by Angajala et al. identified differentially expressed miRNAs associated with the disease [59]. This study compared TCGA miRNA sequencing results from women with QNBC, AR+ TNBC, luminal and Her2+ breast cancers. Hsa-mir-500a, hsa-mir-181a-2, hsa-let-7d, hsa-mir-92a-2, hsa-mir-150, hsa-mir-17, hsa-mir-92a-1, hsa-mir-30a, hsa-mir-210, hsa-mir-455, hsa-mir-130a and hsa-mir-20a were found to be differentially expressed in QNBC with hsa-mir-135b, hsa-mir-18a and hsa-mir-577 being both differentially regulated and correlated with AR expression in QNBC. Our network analysis of these miRNAs reveals common regulatory nodes representing targetable molecules and pathways, including the Myc oncogene and TP53 and PTEN tumor suppressors (Figure 2). All three are regulated by miR-20a-5p, miR17-5p and miR92a-3p at the center of the network. Such miRNAs and their targets and similar analyses may provide novel targetable miRNA networks for the treatment of AR-positive TNBC and QNBC. 

**Figure 3:**
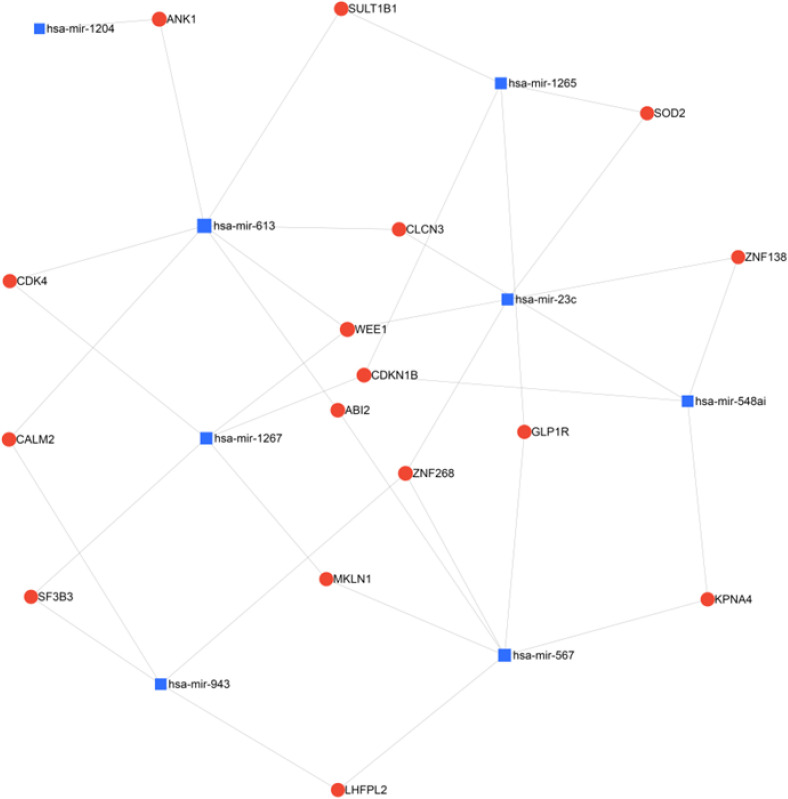
Network analysis of miRNAs found to be upregulated in QNBC by Bhattari et al. using miRNet 2.0 with minimum network filtering. The miRNAs identified by this study and the genes they are known to regulate were analyzed for relatedness and common nodes, revealing prevalent regulation networks and regulated genes in this population.

Recently, Bhattari et al. identified increased copy number alterations (CNA), genomic instability and miRNA dysregulation in QNBC compared with AR+ TNBC [60]. They identified 184 miRNAs that were differentially expressed between these subtypes, with 15 in chromosomal regions with CNA. Expression of eight of these (miR-23c, miR-1267, miR-548ai, miR-613, miR-943, miR-1265, miR-567 and miR-1204) corresponded to the alterations in copy number. This panel was able to discern QNBC from TNBC with a high degree of discrimination. Pathway analysis revealed associations of the 8-miRNA panel with genomic instability, cellular response to DNA damage and cell cycle regulation. Five of these miRNAs were found to be associated with distant metastasis (miR-567, miR548ai, miR-1267 and miR-1265 positively, and miR-23c negatively). Network analysis of the 8-miRNA panel as above demonstrates common nodes involving cell cycle and G2 checkpoint genes CDKN1B and WEE1 (WEE1 G2 checkpoint kinase) (Figure 3). Interestingly, the cyclin-dependent kinase inhibitor gene CDKN1a appeared as a node in network analysis of miRNAs from Angajala et al. above. In addition, notably, the gene encoding Superoxide Dimutase 2, Sod2, appears as a node in networks generated from miRNA panels discovered in both Bhattari et al. and Angajala et al. The SOD2 protein neutralizes reactive oxygen species resulting in anti-apoptotic properties in the context of oxidative stress, inflammation and ionizing radiation [61]. SOD2 is a potential marker of metastatic progression of breast cancer [61]. MAPKs, EGF, Rac, Src signaling pathways are thought to be potentially related to SOD2-associated breast cancer progression [61]. A review also showed an association between reactive oxygen species (ROS), radiation and breast cancer development [62].

## 7. Translational and Clinical Implications of Known Dysregulated miRNAs and Downstream Pathways in QNBC

Research into such miRNA regulatory networks may provide novel targets and allow the selection of targeted therapies based on the prediction of response of AR-positive TNBC versus QNBC to novel and existing therapeutics. Insights provided by this review and the literature cited herein may provide a foundation for the discovery of miRNA and regulated genes and pathways that may be targeted differentially in QNBC and AR+ TNBC, further justifying the stratification of TNBC according to AR expression. One example may be the tumor promoting miR-135b. Since this miRNA is associated with increased proliferation and TGF-β, Wnt and ErbB signaling in basal-like TNBC [58], miR-135b and these pathways may present targetable factors in QNBC. Conversely, introduction of miR-361-5p may be an approach to treating either QNBC or AR+ TNBC in conjunction with AR antagonism. This is a theoretically valid approach given the miR-361-5p inhibition of the EGFR/PI3K/Akt pathway [57], which is relevant in each of these contexts. This approach is supported by evidence of growth and viability inhibition by dual targeting of AR and PI3K in TNBC [63]. Inhibition of this signaling axis may be better suited for AR+TNBC since PIK3CA mutations are ten-fold more prevalent in this subtype than in QNBC [63], although alternate mechanisms of suppression of this pathway, such as through regulation by miRNAs including miR-361-5p may be more prevalent in QNBC. In analysis of the few published studies that identify differentially regulated miRNAs in QNBC versus AR+TNBC, regulation of PI3K/PTEN and Myc signaling, superoxide dismutase and cell-cycle checkpoint come into view as potential pathobiologically meaningful pathways that may be dysregulated by disease-specific miRNAs. The analysis of TNBC subtypes in African American women by Angajala et al. identified differentially regulated miRNAs that regulate Myc, TP53 and PTEN [59]. The use or targeting of these miRNAs and targets may have promise as therapeutic approaches, and PI3K/Akt/mTOR and cell cycle regulators have been previously considered to be prospective therapies for QNBC [15]. In fact, the recent study by Bhattari et al. that identified Copy Number Alteration (CAN)-associated and dysregulated miRNAs in QNBC identified several miRNAs that regulate cell-cycle checkpoint and cyclin dependent kinase regulators, namely miR-23c, miR-548ai, miR-613, miR1267 and miR1265 [60]. Cyclin-dependent kinase inhibitors (CDKIs) can be effective in the treatment of breast cancer, particularly in those with dysregulated PI3K signaling [64]. Both of these pathways may be aberrantly regulated to miRNAs in QNBC. A potential common thread between the few studies may suggest a role for miRNA regulation of SOD2 in QNBC. Although superoxide dismutases are known to promote breast tumor progression and metastasis [61], no direct inhibition of these enzymes has emerged as therapy. Indirect regulation of superoxide dismutases using miRNAs may be achievable while simultaneously targeting other relevant pathways as suggested by our network analysis.

Neoadjuvant therapy is central to therapy for TNBC given increased response rates compared with other breast cancer types [65]. In the context of neoadjuvant therapy for early stage TNBC, anthracycline, taxane and cyclophosphamide are standard, while platinum-based chemotherapy and targeted therapies, including PARP inhibition have been proposed [66,67]. Given the molecular pathways and miRNA-regulated molecules related to AR signaling described above, other targeted therapies should be explored in this setting. These may include mTOR/PI3K inhibition, cell cycle inhibition and androgen blockade for the LAR subtype, and EGFR and PARP inhibition for other TNBC subtypes. While addition of mTOR inhibitors to neoadjuvant chemotherapy did not seem to affect outcomes in a general TNBC population, [12,68] mTOR inhibition did improve objective response rates in mesenchymal TNBC patients with PI3K aberrations [69]. Addition of PARP inhibitor and carboplatin to standard neoadjuvant chemotherapy increased the complete response rate in TNBC [65]. The question of whether regulatory networks specific to TNBC and QNBC can be leveraged to maximize the efficacy of neoadjuvant therapy is compelling. Broad regulation using the histone deacetylase inhibitor valproic acid has in fact been found to synergize with neoadjuvant chemotherapy, CDK inhibitors and PARP inhibitors (PARPi) [70]. The direct use of miRNAs or their targeting to affect such pathways may hold promise in the neoadjuvant setting, but requires further understanding of these regulatory networks.

## 8. Direction of miRNA Research in QNBC

The field of study specific to QNBC is young, leaving much to be discovered about unique pathobiology, regulatory networks, exploitable therapeutic targets and prognostic or theranostic markers. However, there is some understanding of the unique molecular profiles in QNBC. This knowledge can be built on to extrapolate hypotheses regarding the roles of these pathways, their regulation and their targetability with therapeutic interventions. Investigation of miRNA profiles, regulatory networks and pathway analysis of these networks in QNBC compared with AR+ TNBC and other breast cancer subtypes has been sparse so far. Studies using large cohorts comparing these subtypes and additional mechanistic studies of transcript regulation by differentially expressed miRNAs and downstream phenotypic changes relevant to tumorigenesis and progression in QNBC vs AR+ TNBC are needed. Such studies will be required to appreciate the role and potential targeting of these regulators and their regulatory networks in these diseases.

## 9. Conclusions

There is increasing evidence of unique molecular profiles and targetable pathways other than AR signaling in QNBC versus TNBC and other breast cancer subtypes. These differentiators continue to strengthen the justification for AR expression evaluation of TNBC and classification accordingly. While mutational dysregulation of targetable signaling pathways such as PI3K are more readily identified in other subtypes, these pathways may also be dysregulated in QNBC via transcript modulation by miRNAs. Recent studies have identified consistently and differentially dysregulated miRNAs targeting multiple factors that are relevant to breast cancer pathobiology, including Myc, PI3K, TP53, SOD2 and cell cycle checkpoint factors. Further research into QNBC-specific dysregulation of miRNAs, transcripts and molecular and functional networks can enable the exploitation of these unique factors for the targeted treatment in the face of an otherwise intractable therapeutic challenge. 

## Figures and Tables

**Table 1:** Lehmann classification 2011 and 2016. Pathways and molecular targets in TNBC subtypes. Classical molecular and cellular TNBC subtype classifications are listed, along with their distinctive dysregulated signaling pathways and potential molecular targets. Potential targeted therapies associated with classifications and mutations are listed in parentheses. BL, basal-like; IM, immunomodulatory; M, mesenchymal, MSL, mesenchymal stem-like; LAR, luminal androgen receptor.

Expression-Based Molecular Classification						
BL1 (17.9%)	BL2 (11.1%)	IM (21.1%)	M (20.8%)	MSL (6.5%)	LAR (9.2%)	Unstable (13.5%)
**Molecular Targets**						
PARP1,CHEK1, RAD51, PLK1, TTK, AURKA/B	EGFR, mTOR, MET,EPHA2	JAK1/2, STAT,BTK, NFκB,LYN,IRF1	PI3K,IGF1R mTOR, SRC, FGFR PDGFR	PI3K, mTOR, MEK1/2, SRC, IGF1R, FGFR, PDGFR, NFkB	AR, HSP90, PI3K, FGFR4	PARP1, RAD51, PLK1, AURKA/BTTK,CHEK1
**Treatment**						
Antimitotic agents (platinum, PARPi)		TKI,mTORi, eribulin mesylate			Anti-androgen	
**Genetic Mutation-Based Profiling**						
BRCA1/2, PARPi	PIK3CA (PI3Ki)	PD-L1 (Immunotherapy)	CDK4/6 (CDK4/6i)	TP53	PTEN	EGFR
**Expression-Based Cellular Classification**						
Basal-like		Claudin-high		Claudin-low	LAR	
**Androgen Receptor (IHC)-Based Profiling**						
Androgen receptor-positive (Androgen antagonists, e.g., bicalutamide, anzalutamide)				Androgen receptor-negative (Restricted to chemotherapy)		
**Molecular Targets in QNBC**						
Cell metabolism				acyl-CoA synthetase4 (ASCL4)		
Tumor immune microenvironment				Tumor-infiltrating lymphocytes (TIL), Tumor necrosis factor superfamily member 10 (TNFSF10), Programmed death ligand 1 (PD-L1)		
Cell growth and proliferation				EGFR,HER4, CK5/6,CDK6,PTEN,ki-67		
